# Stimulants in Disease

**Published:** 1865-08

**Authors:** 


### Editorial Department.

### Stimulants in Disease.

It has become so fashionable for physicians to recommend stimulants in all cases of disease, all cases of discomfort, all cases where advice is asked, that finally it is taken without consultation, upon the presumption that if it has not, it will be the first remedy recommended by the medical attendant. That all patients require stimulus has well nigh become a conviction in the minds of physicians, and is the other natural extreme from that old and nearly exploded notion that disease was mostly inflammatory in character and required depletion as a remedy. Experiments are made and published to show that "alcohol is food," and as it is an agreeable dish, it has come to stand very high in the list of dietetics. This position is sustained by worthy names, and good physicians are led to believe that alcohol is useful in moderate quantities in health, and of immeasurable and indispensable benefit in disease. This notion has gone to such an extent that even infants are fed on alcoholic stimulants to give them strength, while milk, their natural food and support, is wholly neglected, a panada of wine and some Frenchman's concoction of starch or bran forming the staples of nutriment. Whisky has attained to the dignity of an universal remedy. Coughs of any time standing are treated to a dose of it every four hours; pains in the chest, often from dyspeptic disease, mainly induced by its abuse, are treated by whisky, in "table spoonful doses after meals," as a sovereign remedy for all pectoral derangements. Every body who is weak, every body who is sick, all who are in any way unwell, take whisky as the great all-powerful, all-healing remedy. That stimulants are useful in low forms of disease appears very probable; that those who are from any cause requiring some temporary artificial support may derive benefit from this source is quite certain, but whoever watches the action of stimulants both upon the healthy and diseased, cannot fail to discover that its present indiscriminate adoption for universal purposes is the greatest calamity which has ever befallen, or ever can befall the community. Whisky is a curse in comparison with which all other social vices or evils are blessings; it so infinitely outweighs all other evils in its effects upon the lives and healths of mankind, that it is really the "sum of all evil." If it has benefited one poor consumptive who used it temperately, it has induced fatal disease in a hundred others who have used it improperly; if it has sustained one patient through the stage of collapse or prostration incident to severe disease, it has carried a hundred into a condition of prostration and collapse from which there is no recovery. We have not commenced a Father Mathew crusade in the temperance reformation, but have had opportunity of observing that this whisky medication is becoming alarming; it grows more and more serious every year, and every sober man, medical men in particular, must have anticipated an approaching day of universal debauch, when every body will die drunk.

Not a day passes but these subjects are presented for the consideration of practitioners of medicine, and we solemnly believe that if physicians owe anything to the community, outside their round of professional advice, it consists in a frank, candid, outspoken warning as to the poisonous effects of excessive stimulation. It would be regarded as criminal if any other agent was being used to one-hundredth part the injury—was causing disease and death in anything like the frequency, and physicians said nothing more about it, than they do upon the subject of intemperance. But this is not our point; we are desiring to call attention to the fact that stimulants are too freely and too inconsiderately recommended in mild and unimportant conditions of derangement, and are not reserved for exhaustion, debility or inanition where they can be resorted to with advantage. It seems as though this universal and unlimited stimulation had come to be regarded as harmless, and that even physicians are not conscious of its ill effects. Stimulus is now always prescribed in cases of consumption or suspected consumption, and no doubt in some cases of tuberculosis, stimulants are useful in arresting the disease or in raising the system from a state of debility which favors its progress, but it must be prescribed definitely, and in suitable quantities. If we prescribe opium, we give quantities and intervals; we rarely, if ever, tell a patient to take opium, or morphine, or any other medicine except whisky, without these conditions, but this is a remedy which appears to stand as an all-healing panacea, which in all quantities always does good, and can never do harm. It would not be difficult to present evidence which would be conclusive, that stimulus even in consumption is very generally so used as to favor the progress of the disease it is taken to arrest; that it has caused by its improper use a great many more cases of consumption than it can ever cure; yet this does not hold as argument against its judicious employment when indicated. We would not oppose its proper use, but we protest against its unnecessary recommendation to every body and for almost every thing. That it is used a great deal too much there can be no doubt, and that many cases of disease would terminate in recovery much sooner if less whisky and more food, air, exercise and other hygienic conditions were prescribed in its stead. Stimulus is not curative in the eruptive or continued fevers where it is now so generally used. Typhoid fever will pass through its stages and generally terminate favorably if sustained by food without stimulus; it is quite possible that many cases are protracted by the universal habit of stimulation, even if the cases are mild and require little or no medication. We have not proposed to say a word of its use by people in health, but simply to call upon the profession, the natural guardians of the public health, to avoid when practicable, endorsing an article as useful, which in health is always injurious and is producing untold misery, and in disease, except in rare cases, of doubtful value.

